# Long-Term BOTDA Baseline Assessment of a Fiber Optic Sensing Textile on a Composite Bridge Girder

**DOI:** 10.3390/s26196198

**Published:** 2026-09-30

**Authors:** Guoqiang Cui, Rui Wu, Lidan Cao, Sabrina Abedin, Maryam Abazarsa, Tzuyang Yu, Xingwei Wang

**Affiliations:** 1 Department of Electrical and Computer Engineering, University of Massachusetts Lowell, Lowell, MA 01854, USA; guoqiang_cui@student.uml.edu (G.C.); rui_wu@student.uml.edu (R.W.); lidan_cao@student.uml.edu (L.C.); sabrina_abedin@student.uml.edu (S.A.); 2 Department of Civil and Environmental Engineering, University of Massachusetts Lowell, Lowell, MA 01854, USA; maryam_abazarsa@student.uml.edu (M.A.); tzuyang_yu@uml.edu (T.Y.)

**Keywords:** BOTDA, distributed fiber optic sensing, sensing textile, bridge structural health monitoring, baseline comparability, measurement-path segmentation

## Abstract

**Highlights:**

**What are the main findings?**

- The six-year BOTDA baseline behavior showed clear segment-dependent variability, with larger cross-campaign deviations in the non-structural lead and return segments than in the bonded structural sensing sections.
- The bonded structural sensing sections exhibited lower baseline deviation and narrower drift-rate distributions, indicating better long-term baseline repeatability within the installed sensing path.

**What are the implications of the main findings?**

- Whole-path BOTDA metrics can be strongly influenced by non-structural portions of the sensing path and should therefore be interpreted with caution in long-term bridge monitoring.
- The reported deviation and drift metrics are most suitable for cross-campaign baseline comparison and follow-up anomaly screening, rather than as direct indicators of structural deterioration.

**Abstract:**

This paper presents a six-year assessment of the baseline comparability of a Brillouin optical time-domain analysis (BOTDA)-based sensing textile installed in a composite bridge girder under unloaded field conditions. Six monitoring campaigns collected from 2020 to 2025 were analyzed using campaign-temperature-corrected strain profiles. The known physical segmentation of the sensing path was used to evaluate cross-campaign behavior through segment-wise mean deviation, root-mean-square deviation (RMSD), percentile-based deviation, and position-wise drift-rate metrics. The results show clear segment-dependent variability, with larger deviations occurring mainly in the non-structural lead and return segments than in the bonded structural sensing sections. In the 2025 campaign, the combined bonded structural segments exhibited an RMSD of 292 µε and a 95th-percentile absolute deviation of 508 µε. Their mean drift rate was approximately −11 µε/year, with a 95th-percentile absolute drift rate of approximately 101 µε/year. These results indicate better long-term baseline repeatability within the bonded sensing path than in the non-structural portions of the measurement chain. However, because temperature correction was based on campaign-average surface measurements rather than a distributed temperature profile, residual thermal effects cannot be fully separated from the observed long-term variation.

## 1. Introduction

Distributed optical fiber sensing (DFOS) based on Brillouin optical time-domain analysis (BOTDA) provides long-range and spatially distributed measurements of strain- and temperature-sensitive responses along optical fibers [1,2,3]. These capabilities have enabled applications in bridges, tunnels, pipelines, and other large-scale civil infrastructure, where distributed measurements can provide information that is difficult to obtain using conventional point sensors [4,5,6,7,8,9]. For long-term field monitoring, however, an important challenge is not only whether distributed measurements can be acquired, but also whether baseline profiles collected during different field campaigns can be compared reliably under changing environmental and measurement conditions [10,11].

Textile-integrated optical fiber sensors have been developed to improve fiber protection, handling, routing control, and installation efficiency in large structures [12,13,14]. Our previous work demonstrated the design and field installation of a BOTDA-based fiber optic sensing textile in the Grist Mill Bridge and evaluated its response during controlled truck-loading tests [15]. A subsequent study reported multi-year monitoring results from the same bridge and demonstrated the feasibility of obtaining long-term strain profiles using the installed sensing textile [16]. These earlier studies primarily addressed sensing-textile implementation, field deployment, load response, and descriptive long-term monitoring. They did not quantitatively evaluate cross-campaign baseline comparability, segment-dependent variability, or position-wise long-term drift over the complete sensing path.

In practical DFOS installations, the complete fiber path commonly contains physically distinct regions, including bonded structural sensing sections, lead and return fibers, transition regions, connectors, and other non-structural portions of the measurement chain [17,18,19,20,21,22,23,24,25]. Accurate mapping of the physical sensing path is also important for relating distributed measurements to installed structural regions [23,26]. These regions may respond differently to routing, anchoring, handling, and environmental exposure and therefore should not be interpreted as structurally equivalent. Distinguishing their physical roles is an important prerequisite for reliable interpretation of long-term distributed measurements.

In this study, six periodic no-load BOTDA measurement campaigns collected from 2020 to 2025 were analyzed to quantitatively assess long-term baseline comparability in a field-installed sensing textile. The known physical segmentation of the sensing path was used to evaluate mean deviation, root-mean-square deviation (RMSD), percentile-based deviation, and position-wise drift rate across physically distinct fiber sections. Whole-path results were also compared with those obtained from the bonded structural sensing sections to quantify the influence of non-structural portions of the measurement path and to identify localized regions requiring follow-up attention.

## 2. Materials and Methods

### 2.1. Bridge Girder and Segment Layout

**Figure 1:**
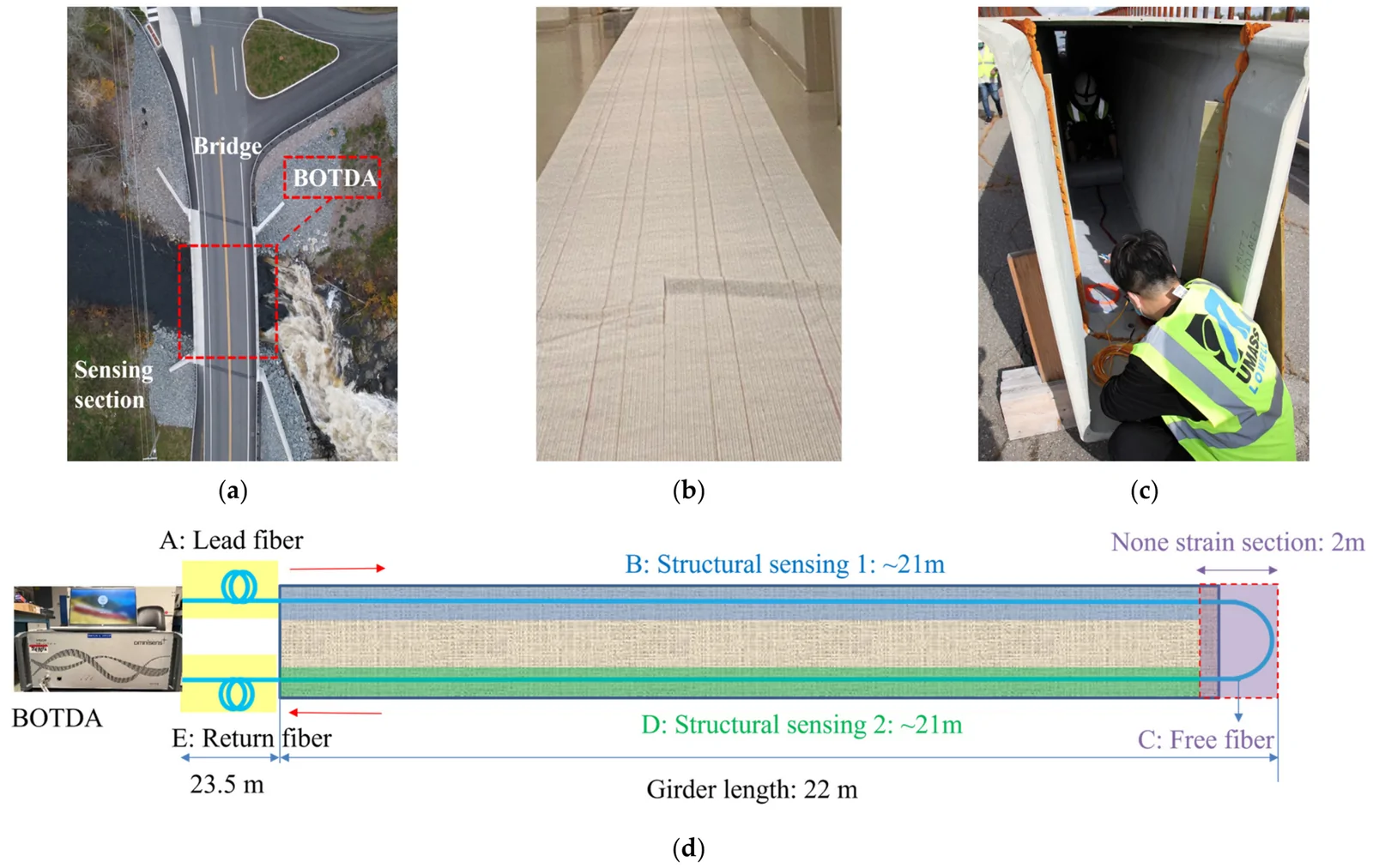
(**a**) Photograph of the Grist Mill Bridge. (**b**) Photograph of the fiber optic sensing textile before installation. (**c**) Photograph of the sensing textile installation inside the bridge girder. (**d**) Schematic of the loop-back sensing layout and segment definitions.

The sensing textile was installed on the Grist Mill Bridge in Hampden, Maine, USA. The bridge has a span of approximately 22.9 m and consists of five fiber-reinforced polymer (FRP) girders supporting a composite concrete deck. The sensing textile was bonded along the interior surface of one girder for distributed strain monitoring. The bridge is exposed to outdoor seasonal environmental conditions, including substantial temperature variations throughout the year, which are relevant to the interpretation of long-term BOTDA baseline measurements. Figure 1a–c show the Grist Mill Bridge, the fiber optic sensing textile before installation, and its installation inside the bridge girder, respectively.

For the segment-aware analysis, the complete measurement path was partitioned into five segments according to the physical installation role of each fiber section and the processed BOTDA distance coordinate. The segment definitions, nominal lengths, and corresponding BOTDA distance intervals are summarized in Table 1. Segment A corresponds to the lead fiber (0–23.5 m), Segment B to structural sensing Section 1 (23.5–44.5 m), Segment C to the free-fiber transition region (44.5–46.5 m), Segment D to structural sensing Section 2 (46.5–67.5 m), and Segment E to the return fiber (67.5–91.0 m). Because of instrument sampling and trimming near the sensing-path ends, the maximum processed distance is slightly below the nominal 91 m path length. The segment boundaries were defined from the as-built sensing layout and the corresponding BOTDA distance coordinates. Because the BOTDA spatial resolution was 1 m, the exact physical transition between adjacent segments could not be localized with sub-meter precision.

Among these five segments, Segments B and D represent bonded structural sensing segments and were therefore used as the primary basis for assessing structural-sensing baseline stability. In contrast, Segments A, C, and E were interpreted as non-structural or transition-path segments, where the measured response may be influenced by lead/return routing, turn-around handling, anchoring conditions, connector effects, or environmental exposure rather than by structural behavior itself. This distinction was used throughout the subsequent analysis to avoid treating the complete BOTDA path as structurally equivalent. A schematic of the loop-back sensing layout and the segment definitions is shown in Figure 1d.

### 2.2. Textile Integration

The sensing textile was fabricated using XP414 laid scrim (Saint-Gobain, Courbevoie, France) as the textile carrier. The sensing fiber was integrated into a smart textile carrier using programmable embroidery, which enabled controlled fiber routing and customized sensing lengths while improving handling during storage, transportation, and installation. During field deployment inside the girder, the textile helped maintain fiber alignment, reduce abrasion risk, and provide more repeatable bonding and anchoring conditions along the bonded structural sensing segments.

Detailed textile design, fabrication, and bridge installation procedures have been reported previously and are therefore not repeated in detail here [11,12,13,14,15,16,17]. In the present study, the textile is considered primarily as a deployment-enabling carrier that supports stable loop-back installation and improves the practical repeatability of the instrumented sensing path, rather than as the main subject of investigation.

### 2.3. BOTDA Principle

BOTDA measures the distributed Brillouin frequency shift (BFS) along an optical fiber by stimulated Brillouin scattering in a pump–probe configuration [27,28,29,30,31,32,33]. The BOTDA measurements were acquired using a DITEST Interrogator UM-031 (Omnisens, Morges, Switzerland). The BFS variation is influenced by both axial strain and temperature and can be expressed as

Δ*ν_B_* = *C_ε_*Δ*ε* + *C_T_*Δ*T* (1)

where Δ*ν_B_* is the Brillouin frequency shift change, Δ*ε* is the strain change, Δ*T* is the temperature change, *C_ε_* is the strain coefficient, and *C_T_* is the temperature coefficient. In this study, both exported BFS and processed strain profiles were retained, while the processed strain profiles were used as the primary basis for long-term baseline metrics because they provide a more direct basis for cross-campaign comparison of structural sensing segments after BOTDA processing. Representative BOTDA acquisition parameters are summarized in Table 2.

A spatial resolution of 1 m was selected as a practical compromise between spatial localization, signal quality, and field acquisition time. Since each bonded structural sensing section was approximately 21 m, this resolution was suitable for the segment-scale comparisons performed in this study. An averaging setting of 500 was used to improve the signal-to-noise ratio and stability of the Brillouin frequency estimation. Given that the measurements were acquired under quasi-static, unloaded field conditions, the increased acquisition time associated with this averaging level did not limit the intended baseline monitoring application.

### 2.4. Campaigns and Temperature

Six field measurement campaigns were annually conducted between 2020 and 2025 under unloaded conditions. The first campaign, acquired on 31 December 2020, was used as the reference baseline for all subsequent cross-campaign comparisons.

During each campaign, the girder-surface temperature was measured using a handheld infrared thermometer at three accessible locations on the underside of the bridge girder. The three measurements were averaged to obtain a representative campaign temperature. The campaign dates and corresponding average surface temperatures are summarized in Table 3.

The average surface temperature was used as the campaign-level thermal input for BOTDA temperature correction. Because these measurements were obtained at discrete surface locations rather than continuously along the sensing path, they do not represent a fully distributed temperature profile. Possible spatial temperature gradients along the girder therefore remain a source of uncertainty in cross-campaign comparison.

### 2.5. Data Processing

A temperature coefficient of 3.5 MHz/°C for the jacketed single-mode sensing fiber in the sensing textile had been calibrated previously and was applied during BOTDA processing [15]. For each campaign, the temperature-related BFS was corrected using the difference between the campaign-average girder-surface temperature and the reference-campaign temperature. Therefore, the exported BFS and strain profiles used in this study represent campaign-temperature-corrected BOTDA outputs rather than raw BFS-only measurements. No second thermal correction was applied during post-processing. Accordingly, the present analysis should be interpreted as an assessment of cross-campaign baseline comparability in campaign-temperature-corrected BOTDA outputs. Any residual thermal mismatch, if present, is treated here as part of the practical field baseline uncertainty rather than as a fully isolated physical-strain component. For this reason, the post-processing focused on baseline referencing, segment-wise stability quantification, long-term drift estimation, and hotspot screening.

For each campaign *i* and position *x* along the processed BOTDA distance coordinate, the baseline-referenced strain difference was defined as

Δ*ε_i_*(*x*) = *ε_i_*(*x*) − *ε_ref_*(*x*) (2)

where *ε_ref_*(*x*) denotes the reference baseline acquired on 31 December 2020. Similarly, the baseline-referenced BFS difference was defined as

Δ*ν_B_*_,*i*_(*x*) = *ν_B_*_,*i*_(*x*) − *ν_B_*_,*ref*_(*x*) (3)

For drift estimation, campaign time was expressed as the elapsed time in years relative to the baseline campaign

∆*t_i_* = *t_i_* − *t_ref_* (4)

where *t_i_* is the campaign time of measurement *i*, *t*_ref_ is the baseline campaign time corresponding to 31 December 2020, and Δ*t_i_* is the elapsed time in years relative to the baseline.

### 2.6. Stability Metrics

To distinguish measurement-path effects from the behavior of the bonded structural sensing sections, all stability metrics were computed both for each individual segment (A to E) and for the combined bonded structural segments (B and D). Segments B and D correspond to the bonded structural sensing sections. Segment C is a short free-fiber transition region. Segments A and E represent lead/return-related non-structural lead and return end-effect sections.

For each post-baseline campaign, the segment-wise mean baseline deviation was ${\bar{\Delta \varepsilon }}_{S}$ calculated as

(5) $${\bar{\Delta \varepsilon }}_{S}=\frac{1}{N_{S}}\sum_{x\in S}\Delta \varepsilon_{i}\left(x\right)\;$$

where *S* denotes the segment (*S* ∈ [*A*,…,*E*]) and *N_S_* is the number of spatial positions within that segment. For brevity, this metric is referred to below as mean Δ*ε* in the figure caption and result discussion.

Repeatability relative to the reference baseline was quantified using the segment-wise root-mean-square deviation (RMSD)

(6) $${\mathrm{RMSD}}_{S}=\sqrt{\frac{1}{N_{S}}\sum_{x\in S}{\left[\Delta \varepsilon_{i}(x)\right]}^{2}}$$

A larger RMSD indicates poorer agreement with the baseline and therefore lower inter-campaign repeatability.

To characterize the upper tail of baseline deviation within each segment while remaining less sensitive to isolated single-point extremes, a percentile-based metric was also used:

*P*_0.95_ = *Q*_0.95_ [∣Δ*ε_i_*(*x*)∣: *x* ∈ *S*] (7)

where *Q*_0.95_ denotes the 0.95 quantile operator, and *P*_0.95_ denotes the 95th percentile of the absolute baseline-referenced strain difference within segment *S*. This metric highlights whether a segment contains a substantial population of elevated deviations, rather than being dominated by only one extreme outlier.

Long-term baseline evolution was quantified position by position by fitting a linear regression of baseline-referenced strain difference versus elapsed campaign time:

Δ*ε_i_*(*x*) = α(*x*) + *β*(*x*) ∙ ∆*t_i_* + *r_i_*(*x*) (8)

where α(*x*) is the intercept, *β*(*x*) is the local drift rate, and *r_i_*(*x*) is the residual term. The fitted slope *β*(*x*), expressed in *με*/year, was defined as the position-wise long-term drift rate. Here, the linear drift rate is used as an empirical descriptor of cross-campaign baseline evolution rather than as a mechanistic degradation parameter. Segment-level drift behavior was then summarized using the distribution of *β*(*x*) within each segment, including the mean, median, *P*_0.95_(∣*β*∣), and maximum ∣*β*∣.

These metrics were interpreted comparatively. A low mean deviation and a low RMSD indicate good baseline consistency, a low *P*_0.95_ indicates limited localized excursions, and a narrow drift-rate distribution indicates better cross-campaign baseline consistency. Positions within the bonded structural sensing segments (B and D) were flagged as hotspot candidates when the magnitude of the local drift rate exceeded the 95th percentile of the drift-rate distribution in the combined bonded structural segments (B and D). These hotspot candidates were treated as screening targets for follow-up attention rather than as direct evidence of structural deterioration.

## 3. Results

### 3.1. Six-Year Baseline Evolution

**Figure 2:**
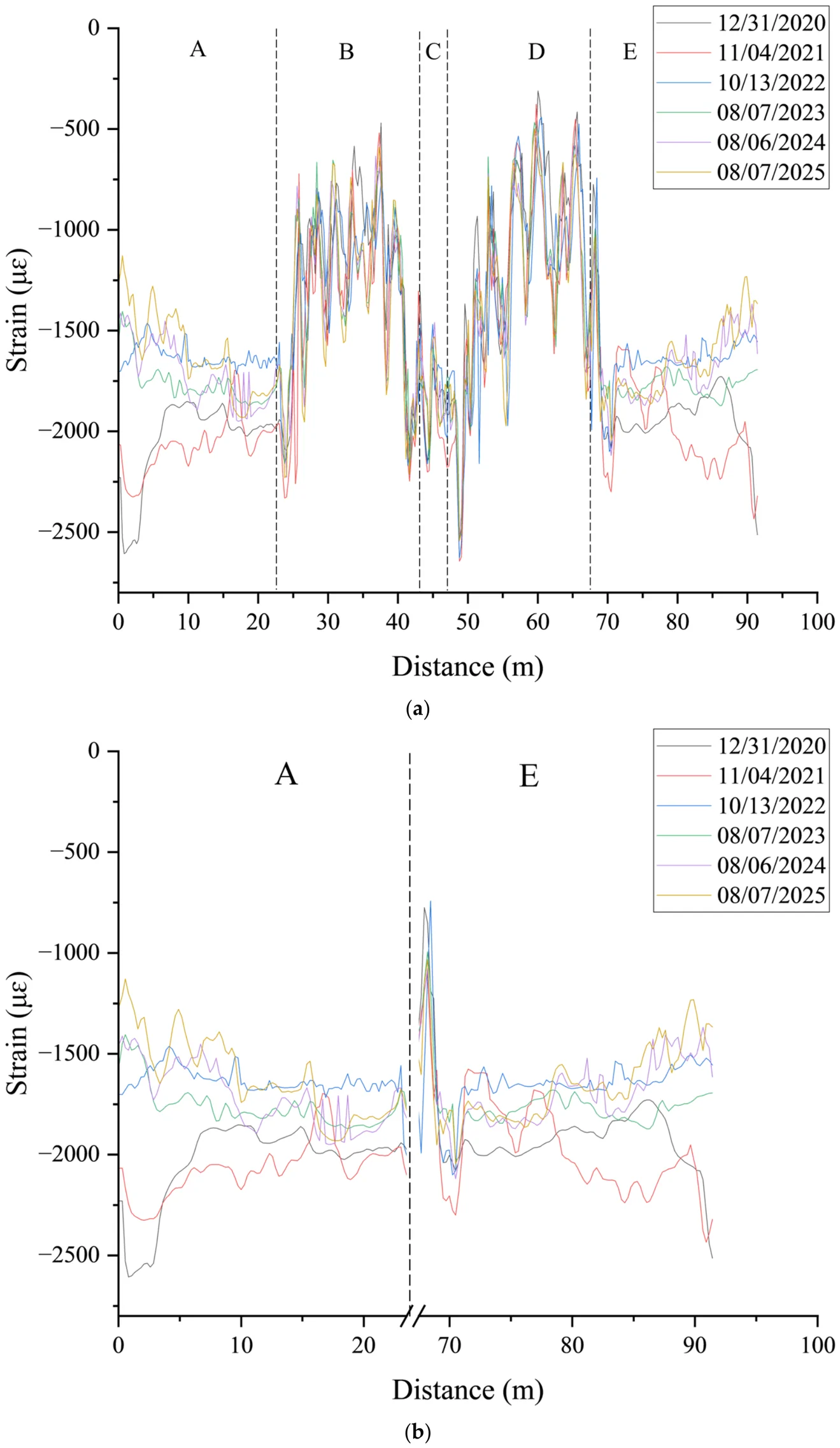

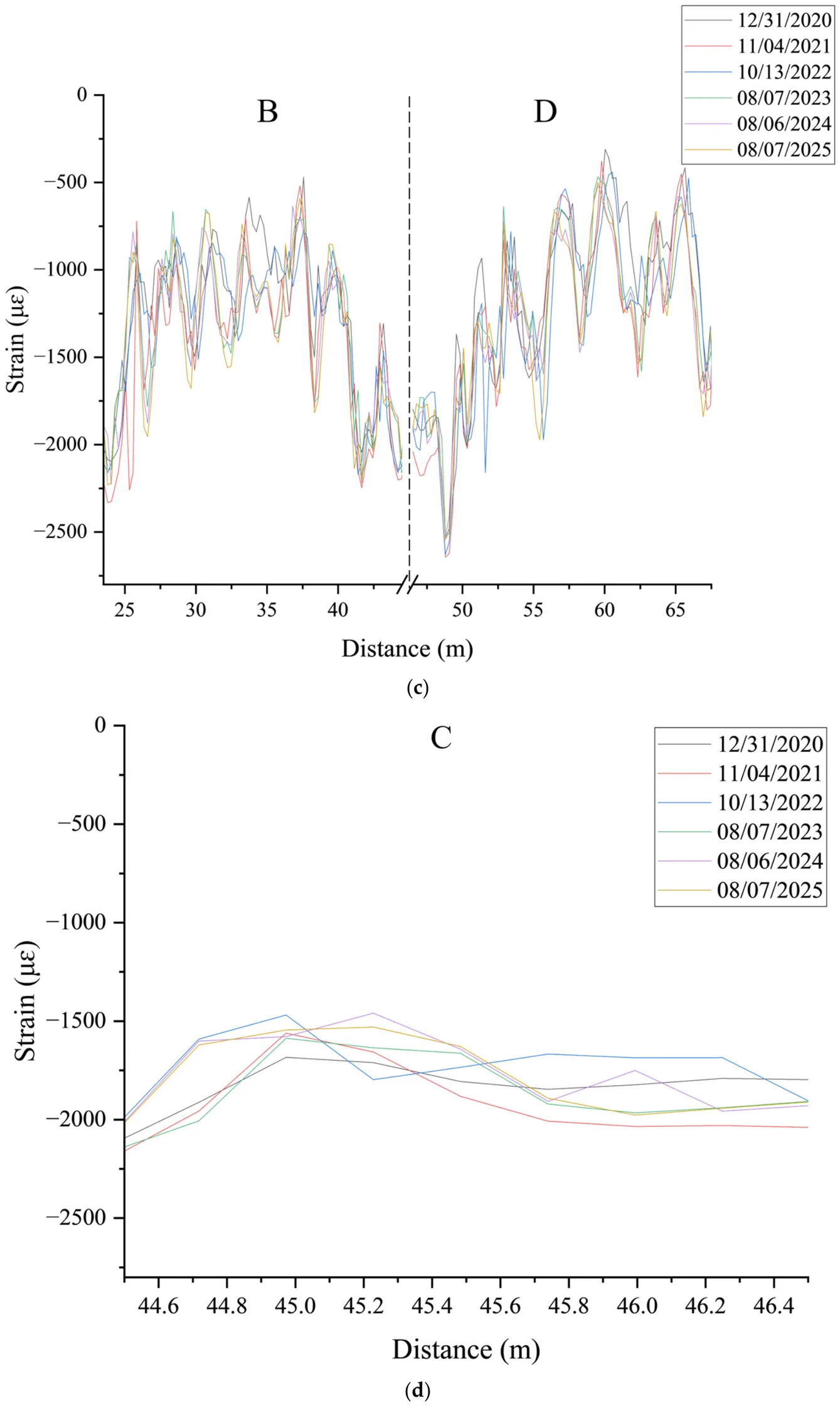
Campaign-temperature-corrected strain profiles across different monitoring campaigns: (**a**) overall view along the full sensing length with segment boundaries (A–E); (**b**) non-structural segments (A and E); (**c**) bonded structural segments (B and D); and (**d**) free-fiber transition segment (C).

Figure 2 compares the campaign-temperature-corrected strain profiles obtained from the six field measurement campaigns along the complete BOTDA measurement path. The profiles exhibit clear spatial nonuniformity across the sensing path. Relatively large inter-campaign variations are observed mainly in the lead and return fiber segments (Segments A and E), whereas the bonded structural sensing segments (Segments B and D) show more closely grouped profiles over the monitoring period.

Figure 2b–d further separate the measurement path according to the physical role of each fiber segment. The lead and return segments exhibit larger profile-to-profile variations than the bonded structural sections, while the short free-fiber transition segment shows localized variation over a limited distance range.

### 3.2. Segment-Wise Baseline Deviation

**Figure 3:**
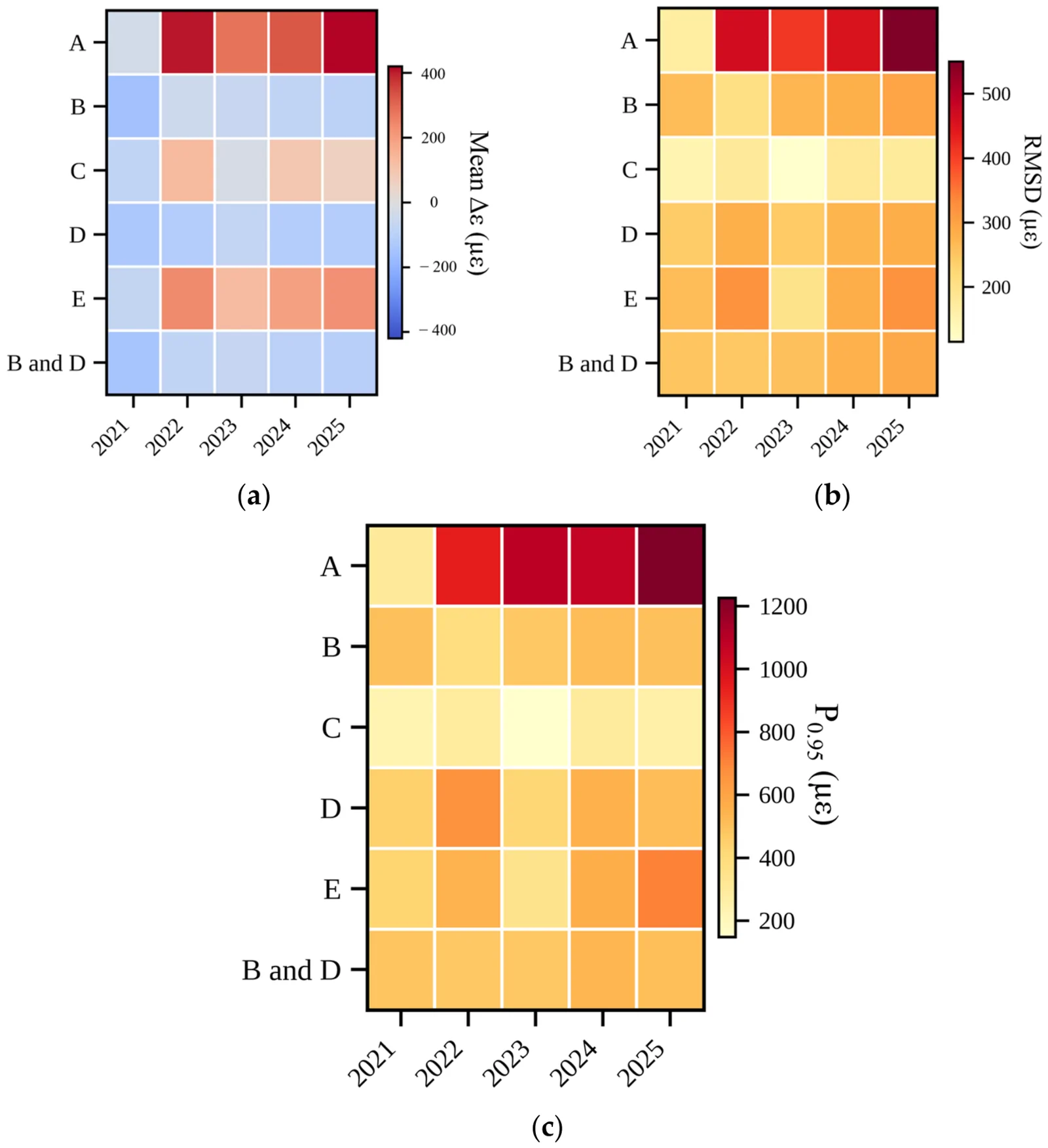
Segment-by-campaign heatmaps of baseline deviation metrics relative to the 2020 reference baseline: (**a**) mean Δε; (**b**) RMSD; and (**c**) *P*_0.95_ (µε). Rows A to E follow the physical segment order, and the final row (B and D) denotes the combined bonded structural sensing segment.

Figure 3 presents segment-by-campaign heatmaps of three baseline-deviation metrics relative to the 2020 reference campaign: mean Δ*ε*, RMSD, and *P*_0.95_ of the absolute baseline-referenced strain deviation. The segment-wise comparison shows that the magnitude of baseline deviation varies substantially along the BOTDA measurement path.

Across the post-baseline campaigns, Segments A and E generally exhibit larger RMSD and upper-tail deviation values than the bonded structural sensing segments B and D. In the 2025 campaign, Segment A reaches an RMSD of 550 με and a *P*_0.95_ of 1226 με, while Segment E shows an RMSD of 325 με and a *P*_0.95_ of 711 με. For the combined bonded structural sensing segments B and D, the corresponding values are lower, with an RMSD of 292 με and a *P*_0.95_ of 508 με.

To quantify the effect of including all portions of the BOTDA path in the baseline assessment, Table 4 compares the 2025 whole-path metrics with those calculated only from the bonded structural sensing segments. The whole-path RMSD is 381.1 με, compared with 292 με for Segments B and D. Similarly, the whole-path *P*_0.95_ is 798 με, compared with 508 με for the bonded structural segments. The mean drift rate changes from approximately −11.2 με/year for Segments B and D to +34.6 με/year for the full measurement path, while *P*_0.95_(∣*β*∣)increases from 101.2 to 177.9 με/year. These comparisons show that whole-path baseline metrics are strongly influenced by the non-structural lead and return sections.

### 3.3. Position-Wise Long-Term Drift

**Figure 4:**
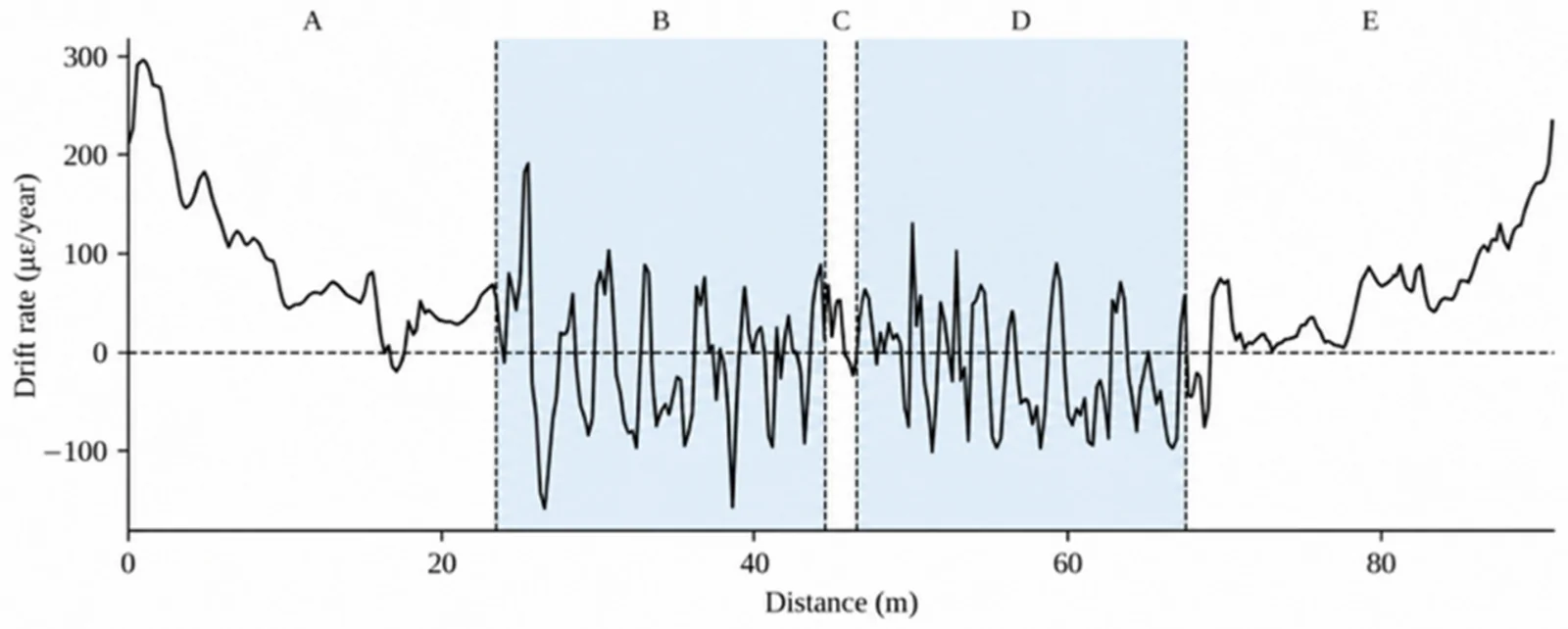
Position-wise long-term drift rate along the complete BOTDA measurement path. Segment boundaries (A to E) are annotated, and the shaded regions indicate the bonded structural sensing segments (B and D).

To characterize long-term baseline evolution along the sensing path, the baseline-referenced strain difference at each BOTDA distance position was regressed against elapsed campaign time. The fitted slope, *β*, was used as a position-wise descriptor of long-term baseline drift. Figure 4 shows the resulting drift-rate profile along the complete measurement path, and Table 5 summarizes the drift-rate statistics for each segment.

The drift-rate distributions differ substantially among the fiber segments. Segment A exhibits the largest positive mean drift rate, approximately +95.3 με/year, together with a *P*_0.95_(∣*β*∣) of 270.3 με/year. Segment E also shows relatively large drift, with a mean of +57.5 με/year and a *P*_0.95_(∣*β*∣) of 167.4 με/year. In comparison, Segment B has a mean drift rate of −7.9 με/year and Segment D has a mean drift rate of −14.5 με/year. When Segments B and D are combined, the mean drift rate is approximately −11.2 με/year and the *P*_0.95_(∣*β*∣) is 101.2 με/year.

Although the bonded structural sensing segments show a narrower overall drift distribution than the lead and return segments, several localized positions within Segments B and D exhibit comparatively elevated ∣*β*∣ values. Based on the screening criterion defined in Section 2.6, the most notable candidate locations occur near 25–27 m, around 38.6 m, and around 50–53 m along the processed BOTDA distance coordinate. These locations are treated as follow-up screening targets rather than direct evidence of structural deterioration.

## 4. Discussion

### 4.1. Structural-Section Stability

The long-term baseline behavior differed among fiber sections because of their different physical roles. The lead and return segments are more susceptible to routing, connector handling, end effects, local anchoring, and environmental exposure, whereas the bonded sensing sections are mechanically coupled to the bridge girder and provide a more direct representation of the structural sensing path.

The lower variability observed in the bonded segments indicates better long-term baseline repeatability of the installed sensing path. However, this should not be interpreted as direct evidence of unchanged structural condition because residual environmental and thermal effects may still contribute to the measured response.

### 4.2. Hotspot Screening

The position-wise drift analysis identified several localized regions within the bonded structural sensing segments with relatively elevated long-term deviation. These locations were treated as follow-up screening targets rather than as direct evidence of structural deterioration.

Because the analysis is based on six periodic field campaigns and campaign-temperature-corrected BOTDA outputs, the identified hotspots may reflect a combination of local sensing-path effects, environmental variability, and possible structural response. Therefore, confirmation would require repeated measurements under comparable conditions, localized inspection, or additional loading tests.

### 4.3. Cross-Campaign Comparability

Cross-campaign comparison of the BOTDA measurements is influenced by both measurement repeatability and environmental conditions. In this study, temperature correction was based on campaign-average girder-surface temperatures measured at three accessible locations rather than on a distributed temperature profile along the full sensing path. As a result, spatial temperature gradients along the girder could not be fully accounted for. The reference campaign was acquired on 31 December 2020, whereas the three most recent campaigns were conducted in August under substantially warmer conditions.

It is also important to distinguish between temperature-induced changes in the optical response of the sensing fiber and thermally induced deformation of the host structure. The BOTDA temperature correction applied in this study compensates for the temperature dependence of the Brillouin response of the silica fiber, but it does not remove actual strain caused by thermal expansion or contraction of the bridge girder. Therefore, the observed cross-campaign baseline differences and apparent long-term drift may reflect a combination of residual optical temperature-compensation error and genuine thermally induced structural deformation.

For this reason, the reported deviation and drift metrics are interpreted as measures of cross-campaign baseline comparability rather than as a complete separation of long-term structural strain evolution from temperature-related effects.

### 4.4. Practical Implications for Long-Term Bridge SHM

For long-term BOTDA bridge monitoring, structural and non-structural fiber sections should be evaluated separately because whole-path metrics can be strongly influenced by lead, return, connector, and routing effects.

The remaining variability within the bonded sensing sections is not negligible. Previous controlled truck-loading tests on the same bridge produced strain responses of several hundred microstrain, which are of the same order as the long-term baseline deviations observed in the present study. Therefore, the reported RMSD and drift metrics are more appropriate for long-term baseline surveillance, cross-campaign comparison, and anomaly screening than for use as direct structural damage thresholds without improved environmental normalization and distributed temperature compensation. The drift-rate values should be interpreted as descriptors of the scale of cross-campaign baseline evolution rather than as annual allowable structural-strain limits.

### 4.5. Limitations and Future Work

This study is based on a single field-instrumented composite bridge girder and six periodic no-load monitoring campaigns rather than continuous measurements. Because each position-wise drift rate was estimated from only six campaign observations, the fitted slope is subject to uncertainty associated with the limited number and timing of the measurements. Therefore, the reported drift should be interpreted as an empirical descriptor of cross-campaign baseline evolution rather than as a statistically precise or mechanistic structural degradation rate.

Another important limitation is the absence of distributed temperature measurements or a dedicated reference fiber along the sensing path. The campaign-average surface temperatures used in this study cannot fully capture spatial temperature gradients along the girder, and residual thermal effects may therefore contribute to the observed baseline differences [23,34]. Future deployments could incorporate a strain-isolated reference fiber for distributed temperature compensation, providing a more rigorous separation of thermal and mechanical contributions [23,34].

The segment boundaries were defined from the known sensing layout and BOTDA distance coordinates. Future deployments could further improve boundary localization using localized thermal tagging after installation, in which a controlled temperature perturbation is used to map physical segment locations to the BOTDA distance coordinate [26].

## 5. Conclusions

This study evaluated the six-year baseline behavior of a BOTDA-based fiber optic sensing textile installed in a composite bridge girder using segment-wise deviation and position-wise drift metrics. The results show that long-term baseline variability is strongly dependent on the physical role of each fiber section, with the non-structural lead and return segments exhibiting larger variations than the bonded structural sensing segments.

For the combined bonded structural segments, the 2025 campaign yielded an RMSD of 292 με and a *P*_0.95_ of 508 με, while the mean long-term drift rate was approximately −11.2 με/year. These results indicate comparatively better baseline repeatability within the bonded sensing path. However, because only campaign-average surface temperatures were available, residual thermal effects cannot be fully separated from the observed long-term variation.

The results therefore support the use of segment-based interpretation for long-term BOTDA field monitoring and highlight the importance of distinguishing sensing-path variability from potential structural change. Localized drift anomalies should be treated as follow-up screening targets rather than direct evidence of structural deterioration.

## Figures and Tables

**Table 1:** Segment definitions used for the segment-aware baseline assessment.

Segment	Role	Nominal Length (m)	BOTDA Distance Interval (m)
A	Lead fiber	23.5	0–23.5
B	Structural sensing 1	21.0	23.5–44.5
C	Free-fiber transition	2.0	44.5–46.5
D	Structural sensing 2	21.0	46.5–67.5
E	Return fiber	23.5	67.5–91.0

**Table 2:** Representative BOTDA acquisition parameters.

Parameter	Value
Sensor configuration	Loop
Sensor length (configured)	100 m
Sample count	392
Spatial resolution	1.0 m
Sampling interval	2.5 ns
Averaging	500
Pulse rate	25 kHz
Frequency start	10.65 GHz
Frequency stop	10.95 GHz
Frequency step (representative)	1.0 MHz

**Table 3:** Campaign dates and averaged girder-surface temperatures.

Campaign (*i*)	Date	Average Girder-Surface Temperature (°C)
1	31 December 2020	−0.1
2	4 November 2021	3.0
3	13 October 2022	12.6
4	7 August 2023	24.7
5	6 August 2024	26.7
6	7 August 2025	26.0

**Table 4:** Comparison of whole-path versus structural-section baseline metrics for the 2025 campaign.

Region	RMSD (µε)	*P*_0.95_ (µε)	Mean *β* (µε/Year)	*P*_0.95_(|*β*|) (µε/Year)
Whole path (A to E)	381.1	798	34.6	177.9
Bonded structural segments (B and D)	292	508	−11.2	101.2

**Table 5:** Drift-rate distribution statistics by segment.

Segment	Mean *β* (µε/Year)	Median *β* (µε/Year)	*P*_0.95_(|*β*|) (µε/Year)	Max(|*β*|) (µε/Year)
A	95.3	63.2	270.3	294.7
B	−7.9	−5.4	140.9	190.6
C	21.8	16.2	62.7	67.3
D	−14.5	−24.9	97.3	130.0
E	57.5	57.7	167.4	233.2
B and D	−11.2	−15.8	101.2	190.6

## Data Availability

The original contributions presented in this study are included in the article. Further inquiries can be directed to the corresponding author.
